# Annotating the microbial dark matter with HiFi-NN

**DOI:** 10.1016/j.isci.2025.112480

**Published:** 2025-04-18

**Authors:** Gavin Ayres, Geraldene Munsamy, Michael Heinzinger, Noelia Ferruz, Kevin Yang, Bastiaan Bergman, Philipp Lorenz

**Affiliations:** 1 Basecamp Research Ltd., London, UK; 2 School of Computation, Information, and Technology (CIT), Department of Informatics, Bioinformatics & Computational Biology, TUM (Technical University of Munich), Munich, Germany; 3 Centre for Genomic Regulation, Barcelona, Spain; 4 Microsoft Research New England, Cambridge, MA, USA

**Keywords:** Microbiology, Computer science

## Abstract

The accurate computational annotation of protein sequences with enzymatic function remains a fundamental challenge in bioinformatics. Here, we present HiFi-NN (Hierarchically-Finetuned Nearest Neighbor search) which annotates protein sequences to the 4th level of Enzyme Commission (EC) number with greater precision and recall than state-of-the-art deep learning methods. Furthermore, we show that this method can correctly identify the EC number of a given sequence to lower identities than BLASTp. We show that performance can be improved by increasing the diversity of the lookup set in both sequence space and the environment the sequence has been sampled from. We proceed to show that we can correct specific mis-annotations in the BRENDA enzymes database reproducing results found by others. Finally, we use HiFi-NN to annotate functional dark-matter protein sequences from NMPFamDB. Our findings pave the way for more accurate functional annotation *in silico*, especially for proteins from distant sequence space.

## Highlights

• Hierarchy aware contrastive learning enables accurate retrieval of related enzymes
• A diverse set of proteins from a global metagenomics pipeline improves performance

## Graphical abstract

**Figure:**
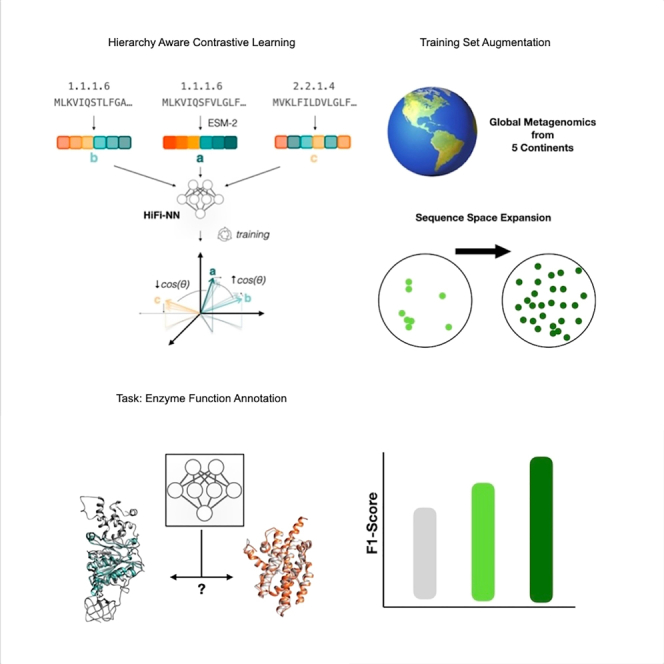

## Introduction

Enzymes are efficient catalysts capable of accelerating chemical reactions by several orders of magnitude.^1^ They play a crucial role in a myriad of processes within living organisms, encompassing functions from respiration and digestion to facilitating muscle and nerve activity. Sequence databases are experiencing unprecedented growth, providing an increasing number of enzymatic sequences that span a wide range of microbial genomes.^2^^,^^3^ While these developments have led to impressive success in training unsupervised models,^4^^,^^5^ a substantial portion of this sequence space remains functionally and taxonomically unannotated and it has been termed the “microbial dark matter” (MDM).^6^^,^^7^ At least one-third of microbial proteins cannot be annotated by aligning them with functionally characterized sequences, and recent studies on the entire AlphaFold database provide evidence that up to 34% of the protein space qualifies as dark matter.^8^ Enzymes are exceptionally attractive in biotechnology, catalyzing a wide array of chemical reactions under mild, non-toxic conditions.^1^ Given the vast potential within the MDM, it is imperative that we develop innovative methodologies for more accurate and cost-efficient enzyme sequence annotation.

The catalytic function of enzymes is commonly annotated with Enzyme Commission (EC) numbers, categorized mainly into oxidoreductases, transferases, hydrolases, lyases, isomerases, ligases, and translocases.^9^^,^^10^ EC numbers are an effective proving ground for functional annotation methods. This is because the EC number describes a reaction catalyzed by a protein, through convergent evolution different folds can catalyze the same reaction,^1^^,^^9^^,^^10^ and so annotation methods which rely solely on sequence homology may fail to generalize to novel folds or sequence motifs. Additionally, a given protein may have multiple EC numbers. A total of 8,243 EC numbers have been cataloged in the BRENDA^10^ database. The numbering system is structured in a hierarchical manner, with 7 top level categories. Each level of the hierarchy denotes a more specific type of reaction than the previous. For example, all carbonic anhydrases (EC:4.2.1.1) are hydrolyases (EC:4.2.1) and that all hydrolyases are lyases (EC:4).

Several computational methods have been developed to annotate EC numbers from amino acid sequence alone, such as the sequence homology–based DIAMOND BLASTp,^11^ or methods based on curation of protein families or sequence profiles,^12^^,^^13^^,^^14^ as well as deep learning methods that were developed more recently. These include DEEPre,^15^ DeepEC,^16^ and CLEAN.^17^ The latter is considered the current state-of-the art deep learning method for predicting EC numbers from sequence. Despite the aforementioned advances in deep learning–based enzyme functional annotation and language models being built to understand the language of life for the bacterial and archaeal kingdoms, comprehensively annotating the MDM remains a challenge.^18^^,^^19^ To address this challenge, we present Hierarchically-Finetuned Nearest Neighbor search (HiFi-NN). HiFi-NN is based on contrastive learning, which has been applied to various protein sequence–related tasks.^17^^,^^20^ The method poses EC number annotation as a retrieval problem. We show that once we have trained a model to learn a metric space wherein sequences with similar function are “close” together the performance of the annotation step can be improved by embedding a larger, more diverse set of sequences and using these as the lookup set for annotation.

HiFi-NN serves as a method by which a query amino acid sequence can be compared to a set of protein sequence embeddings to find those most similar to each query. To this end, we provide a model that has been trained using contrastive learning to map ESM-2 650M^4^ embeddings to a new feature space where distances between the embeddings of sequences correlate to the similarities of their respective EC numbers. The contributions of our manuscript are 2-fold: (1) We develop a method that can correctly identify the EC number of sequences below the twilight zone by incorporating the inherent hierarchy in EC numbers in our contrastive loss, surpassing current state-of-the-art methods. (2) We show that the annotation performance can improve by increasing the sequence and environmental diversity of the lookup set from our proprietary database.

## Results

**Figure 1:**
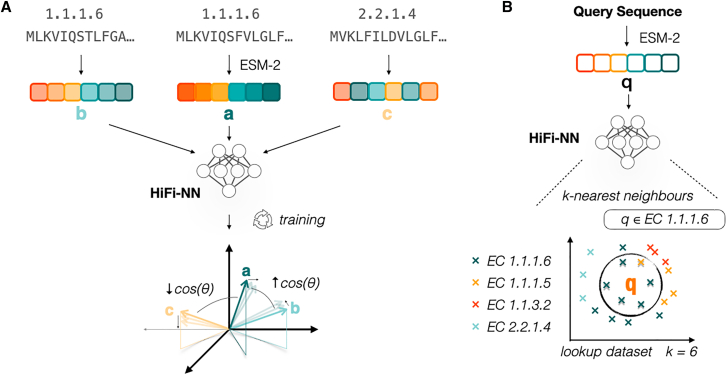
HiFi-NN training protocol (A) ESM-2 650M embeddings of protein sequences with a known EC number are mapped to a new 512 dimensional space where the cosine similarity between embeddings reflects the similarity between their labels. (B) At inference the ESM-2 650M embedding of a query sequence is passed through HiFi-NN and searched against a pre-embedded lookup set. The annotations of the k-nearest neighbors are then transferred to the query sequence.

**Figure 2:**
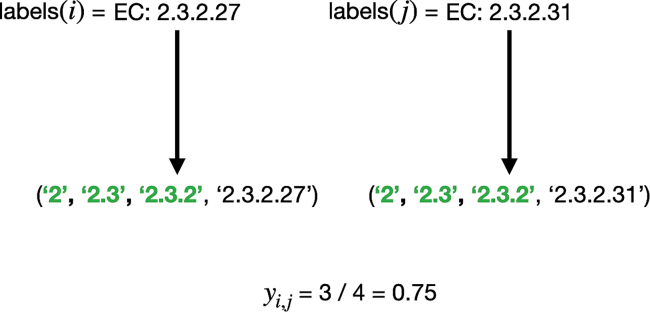
HiFi-NN loss calculation Illustration of the label representation and similarity measure used in the HiFi-NN loss function.

**Figure 3:**
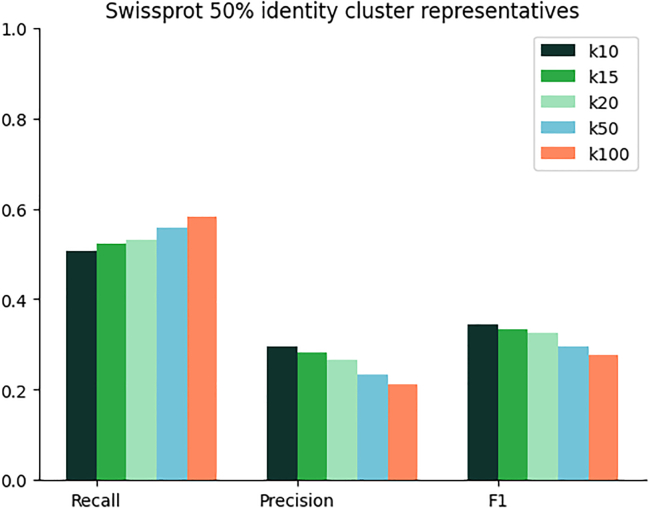
Performance of model as a function of the *k* nearest neighbors retrieved

We train HiFi-NN using contrastive learning, with the training objective to learn an embedding space where the cosine similarity between data points represents the similarities between their functionalities (EC classes) (Figure 1). In particular, for all samples in each batch in our training set, we compute both the pairwise cosine similarities between the model representations of each sample and the overlap coefficient between their associated sets of labels (see additional resources) and minimize the mean squared error between these two quantities (Figure 1A). We leverage the inherent hierarchy of the EC classification system by splitting each EC number into a set with four elements, illustrated in Figure 2. At inference, EC labels are assigned to a query sequence by applying k-nearest neighbor to a pre-embedded lookup table (Figure 1B). We benchmark our method against DIAMOND BLASTp at varying sequence identities to the training set as well on a time-based split of Swiss-Prot. We benchmark against other deep learning methods on a test set of sequences used by both ProteInfer^21^ and CLEAN.^17^ In each instance we use k=20 nearest neighbors when annotating. The performance of our method as function of the k-nearest neighbours is outlined in Figure 3.

### Performance relative to DIAMOND BLASTp

**Figure 4:**
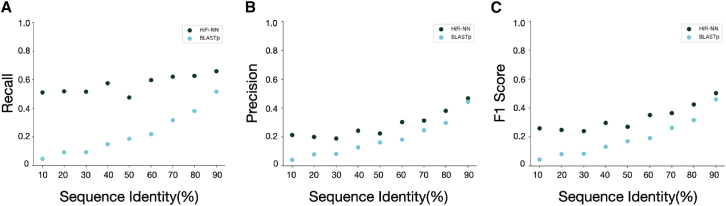
Performance of HiFi-NN compared to DIAMOND BLASTp (A) Recall, (B) precision and (C) F1 score of HiFi-NN vs. DIAMOND BLASTp at varying sequence identity thresholds. We report the sample average of each metric due to the differing test set sizes.

To compare the performance of our method to DIAMOND BLASTp at varying sequence identity thresholds, we clustered the set of sequences from the Swiss-Prot database which have been assigned EC numbers, a total of 274,501 sequences, to sequence identities ranging from 10% to 90% in increments of 10. Our results showed that HiFi-NN outperforms DIAMOND BLASTp at almost all identity ranges in recall, precision and F1 score (Figures 4A–4C), particularly excelling at the low identity range (10–50%). The construction of these datasets is outlined in the Methods section dataset construction and the composition of which is detailed in Table 1. We use the training set for HiFi-NN as the lookup set and it is the same as that for DIAMOND BLASTp in each comparison.

**Table 1:** Size of test set for each clustering identity threshold with the number of EC numbers these sets cover

% Identity Clustering	# Sequences in test set	# EC numbers
10	218	129
20	216	127
30	207	133
40	200	138
50	195	157
60	202	141
70	197	144
80	197	143
90	212	167

This dataset is used to compare HiFi-NN to DIAMOND BLASTp at various sequence percentage identities to the training set.

### Annotating benchmarking datasets outside public databases

We compare our method to other state-of-the-art deep learning protein function annotation tools as well as the current most widely used annotation tool, DIAMOND BLASTp, on the Price dataset.^22^ This dataset was introduced for benchmarking the performance of EC number annotation for the ProteInfer model^21^ and has been used for comparing the performance of multiple other competing deep learning tools since.^17^ It is composed of 149 sequences covering 56 EC numbers. As shown in Table 2, HiFi-NN outperforms DIAMOND BLASTp and other deep-learning annotation methods in recall and F1-score for the task of annotating the Price enzyme dataset.^22^ The training and lookup set we use for this task is Swiss-Prot clustered to 30% sequence identity with 100 cluster representatives removed as a validation set.

**Table 2:** Recall, precision and F1 scores on a dataset of enzymes referred to as the Price-149 dataset.

Method	Recall	Precision	F1-score
ECPred	0.0197	0.0197	0.0197
DEEPre	0.0403	0.0415	0.0386
DeepEC	0.0724	0.1184	0.0846
ProteInfer	0.1382	0.2434	0.1662
ProteinVec	0.2961	0.4901	0.3378
DIAMOND BLASTp	0.3750	0.5083	0.3852
DeepECtransformer	0.3026	0.5263	0.3511
ESM2-650M	0.382	0.510	0.398
CLEAN	0.4671	0.5844	0.4947
HiFi-NN	0.6974	0.62139	0.6162

The trade-off between recall and precision is discussed in the supplemental information. As in Cui et al.^17^ we report the weighted average of each metric to account for class imbalance. The scores from each competing method are as reported in Cui et al.^17^

### Increasing the diversity of the embedding space

**Figure 5:**
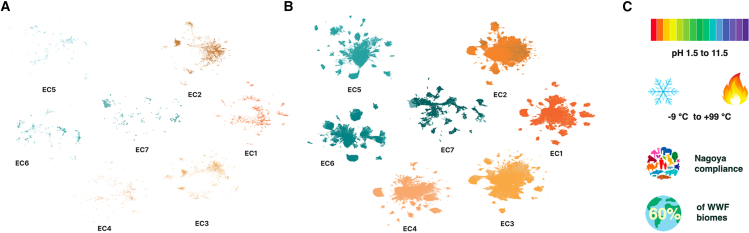
UMAP of Datasets Lookup sets: Swiss-Prot and sequence supplementation from an in-house metagenomic knowledge graph (A) UMAP of Swiss-Prot sequences with an EC number used for training HiFi-NN. (B) UMAP with sequences from (A) overlayed with 2 million sequences from an in-house metagenomic knowledge graph. (C) Key features of the knowledge graph ensuring diverse sampling origin and Nagoya compliance,^23^ from which the subset of 2 million sequences shown in (B) were derived.

**Figure 6:**
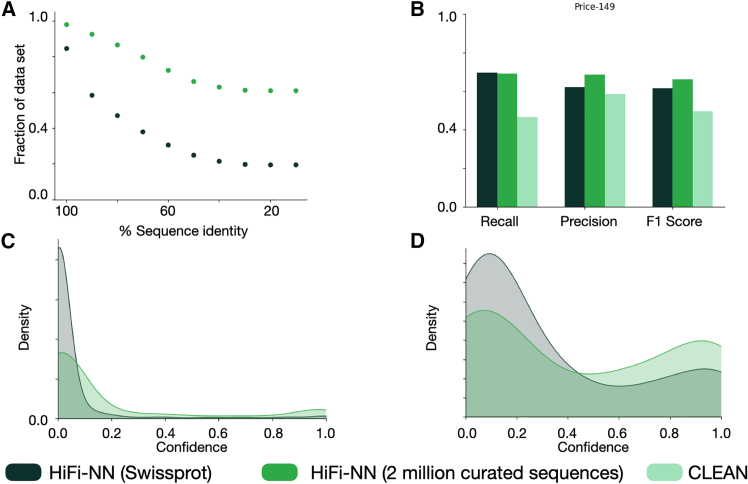
Performance of HiFi-NN on Price-149 (A) The fraction of each dataset retained as we cluster to various sequence identities. In dark green we have Swiss-Prot and in the lighter green we have the 2 million curated sequences from our proprietary metagenomic database. (B) Performance of HiFi-NN on the Price-149 dataset, we see performance is improved by increasing the size of the lookup database without the need for retraining the model. (C) The distribution of estimated probabilities in each *incorrect* annotation on the Price-149 dataset for each lookup dataset. (D) The distribution of estimated probabilities on each *correct* annotation on the Price-149 dataset. We see that the addition of sequences to the lookup set leads to increased confidence in the correct predictions of HiFi-NN.

Seeing that HiFi-NN outperforms DIAMOND BLASTp especially at the low sequence identity range (compared to the lookup datasets), we wanted to test the performance on benchmarking datasets comprising enzyme sequences outside public databases. To that end, we hypothesized that our model would further benefit from supplementing the lookup dataset with sequences from diverse and under-studied environments (Figures 5A and 5B). For this, we use a proprietary Nagoya-compliant^23^ metagenomic knowledge graph, covering broad pH, temperature, and biome ranges (Figure 5C). A curated subset of just over 2 million sequences (Figure 5B) from this knowledge graph was added to the lookup set for annotation. In Figure 6A, we see that the additional sequences are much less redundant than Swiss-Prot.

**Figure 7:**
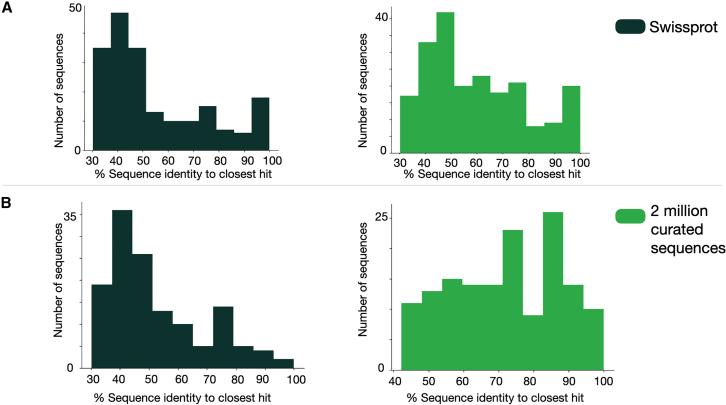
Performance of HiFi-NN as function of training set diversity (A) The sequence identity of each sequence in the Swiss-Prot time-based split test set to its closest hit in each training set. (B) The sequence identity of each sequence in the Price-149 test set to its closest hit in each training set.

**Figure 8:**
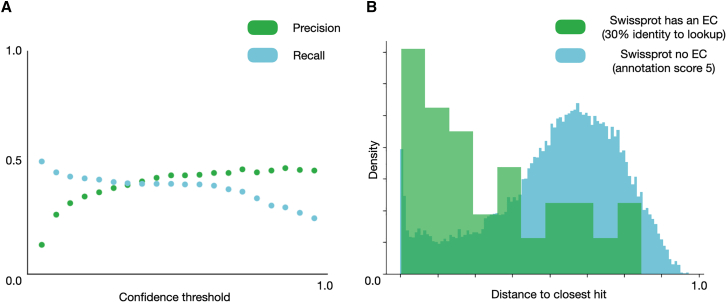
Recall and precision of HiFi-NN as a function of the confidence threshold and distance cutoff selection for making predictions (A) Here, we see the precision and recall of HiFi-NN as we vary the probability threshold. (B) In green we see the distance to closest hit of each sequence in the Swiss-Prot clustered to 30% validation set. In blue we have the distance to the closest hit to each sequence Swiss-Prot which has no EC number and an annotation score of 5. We see that false positives are almost unavoidable, however the tradeoff between false positives and negatives can be controlled by using a distance threshold on predictions. The distance cutoff we use when we refuse to annotate is the 75th percentile of distances to the test set.

To curate the set of sequences which we would add to our lookup set, we first sampled 3 million sequences from our database which had already been annotated with DIAMOND BLASTp such that the sampled sequences would have equal distribution across all EC numbers. To ensure we keep only sequences for which we have high quality labels we annotate them with both DIAMOND BLASTp and HiFi-NN. We then keep only the sequences and annotations for which both methods agree. This gives us 2,089,659 sequences spanning the full set of EC numbers contained in Swiss-Prot, 5,703 at the time of writing. When the lookup dataset is supplemented with these 2 million curated sequences from our in-house database, HiFi-NN outperforms all the aforementioned methods in recall, precision, and F1-score, including HiFi-NN trained on Swiss-Prot only, as outlined in Figure 6B. Crucially, the sequence supplementation to the lookup set also increases the confidence score of correct annotations, Figure 6D. The effect of the confidence threshold is further elaborated on in additional resources. We can see in that the sequences we supplement our lookup set with are closer in sequence space to the Price-149 test set. This could offer a simple explanation for the improved predictive power of the model on this test set (Figures 7 and 8).

### Time based split

**Figure 9:**
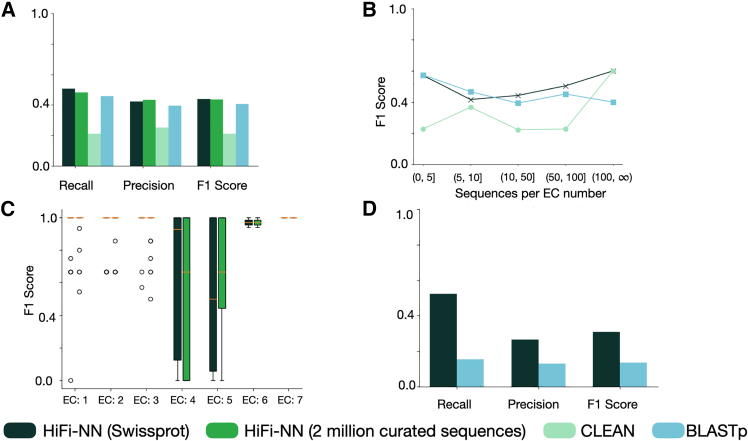
Performance of HiFi-NN on Swiss-Prot (A) Performance of each method on a time-based split of Swiss-Prot. This dataset consists of 244 sequences spanning 135 EC numbers. (B) The F1 score vs. the number of sequences representing each EC number in the HiFi-NN training set. (C) Each boxplot denotes the distribution of F1 scores across each sub class within the 7 top level EC numbers. (D) Performance of HiFi-NN and DIAMOND BLASTp on a larger test set, formed by clustering Swiss-Prot to 50% identity, to see if the performance differences remain consistent across test sets.

To test how HiFi-NN performs in the context of new sequences arriving to a database, we decided to construct a test set from a time based split of Swiss-Prot. Specifically, we constructed a set of sequences which have been added to Swiss-Prot since July 31, 2023, which is the cutoff date of our training set. At the time of writing this is a set of 244 sequences spanning 135 EC numbers. We can see in Figure 9A) the addition of a diverse set of metagenomic sequences does not lead to substantial performance improvements. In addition, DIAMOND BLASTp performs quite well on this dataset. We use the same benchmarking set up as before where we run DIAMOND BLASTp against the HiFi-NN training set to ensure a fair comparison. In Figure 9B, we bucket the test set by the number of sequences representing each EC number in the HiFi-NN training set. We see that for EC numbers which have many representatives in Swiss-Prot, both HiFi-NN and CLEAN perform comparably. Notably, HiFi-NN performs quite well on sequences which have few examples in the training set. We believe this is due to framing the problem as one of retrieval, as it is posed when we use DIAMOND BLASTp. In Figure 9C, we see that the distribution of F1 scores for each sub class within the 7 top level EC numbers are roughly similar for both lookup sets. Further studies could investigate the relationship between performance and the diversity of sequences which represent the class. In Figure 9D, we benchmark HiFi-NN against DIAMOND BLASTp on a larger test set to see if the performance differences remain consistent. To construct the dataset we cluster Swiss-Prot to 50% identity and use 3000 cluster representatives as the test set. We then ensure that each EC number which exists in the test set has at least a single representative in the training set. This gives us a final test set size of 1,977 sequences covering 644 EC numbers and a training set size of 166,404 sequences covering 2,808 EC numbers (≈ 49% of the total number within Swiss-Prot). The details of the dataset construction are outlined in additional resources. Here, we see results consistent with those shown in Figure 4, HiFi-NN outperforming DIAMOND BLASTp significantly.

### Mis-annotations in BRENDA

Rembeza and Engqvist^24^ conducted an experimental investigation of the members of the EC class 1.1.3.15 within the BRENDA database. Based on screening 122 representative sequences from the class they inferred that at least 78% of the class were incorrectly annotated. They also showed that mis-annotations increased over time by examining the relative decline in the canonical flavin mononucleotide (FMN) dehydrogenase domain in members of the class, leading the authors to hypothesize that this number rose to at least 87% of the class being incorrectly annotated.

We decided to annotate the same datasets, sequences taken from BRENDA in 2017 and 2019, to see if our tool could recapitulate their findings. We find that 86% of sequences from the 2017 dataset were not annotated with their expected EC number, 1.1.3.15, and 90% from the 2019 dataset lacked this annotation.

Failing to annotate the supposedly incorrect EC number does not necessarily mean that the annotations we assign the sequences in its stead are correct. Rembeza and Engqvist^24^ suggest some alternative functions for the erroneously annotated sequences. To this effect they use HAMAP^25^ and eggNOG^8^ to annotate the sequences within the class. They note that in this instance only a subset of the sequences are given any annotation, 74% by HAMAP and 59% by eggNOG.

We list the hypothesized alternative functions along with the quantities which HAMAP and eggNOG annotated as compared to the amount annotated by HiFi-NN in Table 3. HiFi-NN annotates all of the sequences in the class, a total of 1,411 sequences.

**Table 3:** Number of sequences annotated with each hypothesized function by each method

Hypothesised function	HAMAP	eggNOG	HiFi-NN
L-lactate dehydrogenase (EC 1.1.2.3)	241	79	5
D-lactate dehydrogenase activity (1.1.2.4)	0	69	378
S-2-hydroxyacid oxidase activity (EC 1.1.3.15)	0	292	196
L-hydroxyglutarate dehydrogenase activity (EC 1.1.99.2)	685	2	684
Glycerol-3-phosphate dehydrogenase activity (EC 1.1.5.3)	0	54	70

Numbers reported from Rembeza and Engqvist.^24^

### Annotating the NMPFams database

**Figure 10:**
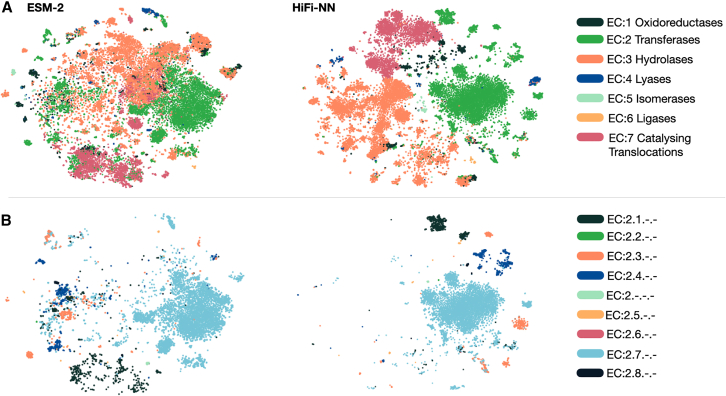
Annotating the microbial dark matter We annotate the set of consensus sequences from the NMPFAMs database with HiFi-NN. We use a distance cutoff of 0.38 and a confidence threshold of 0.5. (A) On the left we have the t-SNE of the ESM-2 650M embeddings of the consensus sequences from the NMPFAMs database and on the right the HiFi-NN embeddings of this same set of sequences. We color each sequences with its predicted top level EC number. (B) Here we focus the set of transferases, EC: 2.-.-.-, from the above t-SNE and highlight based on the associated second level EC number. As we see in both cases the HiFi-NN embeddings cluster into distinct groups reflecting their predicted functional similarity.

The NMPFAMs database^26^^,^^27^ is a collection of metagenomic protein clusters with no or weak hits to Pfam or Reference genomes, which could therefore be considered a suitable dataset for testing the application of computational methods to the task of annotating the MDM. We choose the consensus sequences from this database which corresponds to 106,198 sequences. We annotate using HiFi-NN with a distance cutoff of 0.38 as established in additional resources. The distance cutoff ensures that we do not make a prediction for sequences which are very different from the lookup set. We plot a t-SNE of the embeddings of these annotated sequences, colored by their annotations in Figure 10 alongside a t-SNE of the ESM-2 650M embeddings of the same sequences. We see that the HiFi-NN embeddings cluster together better than their associated ESM-2 650M embeddings and that all members of a distinct cluster of HiFi-NN embeddings are annotated with the same EC number by HiFi-NN.

## Discussion

Here, we develop and benchmark the performance of a new deep learning model for enzyme annotation, HiFi-NN. Our model is a contrastive deep learning–based method that differs from previous models in 2 key aspects: (1) We develop a contrastive loss which allows us to use the EC annotation system and its inherent hierarchy as a natural augmentation method and (2) the annotation performance can be improved without re-training by increasing the size of the lookup set, which we supplement with microbial sequences sampled from diverse environments. It outperforms the current most widely used annotation tool in bioinformatics, DIAMOND BLASTp, as well as all other deep learning tools, when assessed with a microbial enzyme benchmarking dataset.^22^ Furthermore, we have proposed a general approach to tackling the problem of function annotation which is similar in spirit to methods currently in use, i.e., retrieval based, and a loss function which can be adapted to other sets of annotations a protein may have. We then use the model to show we can identify mis-annotated sequences in the BRENDA database. Finally, we use HiFi-NN to annotate a portion of the functional dark matter consensus sequences from the NMPFams database.

### Limitations of the study

We acknowledge that the results presented in this study are quantitative measures from *in-silico* benchmarks and we do not have wet lab validation for instances where the predictions of our method differ from those of other *in-silico* tools. As our method depends on nearest neighbor search and storing embeddings for the entirety of the lookup set, scaling to very large datasets may take significant computational resources.

**Figure 11:**
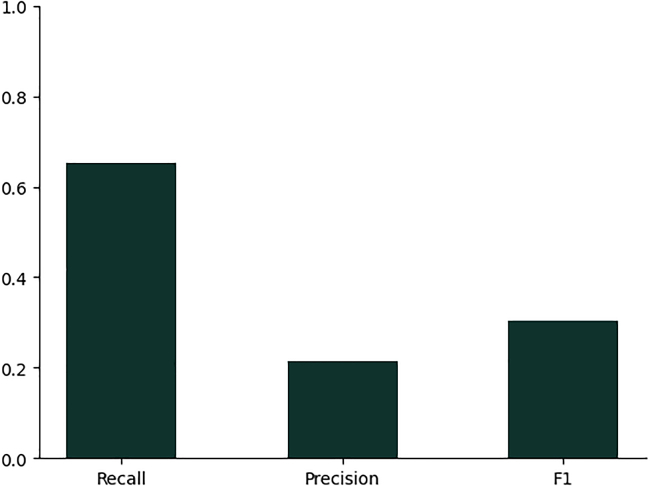
Recall, precision and F1 score on the third level of EC number for sequences which have no 4th level representation in the training set The reported scores are averaged per sample.

**Figure 12:**
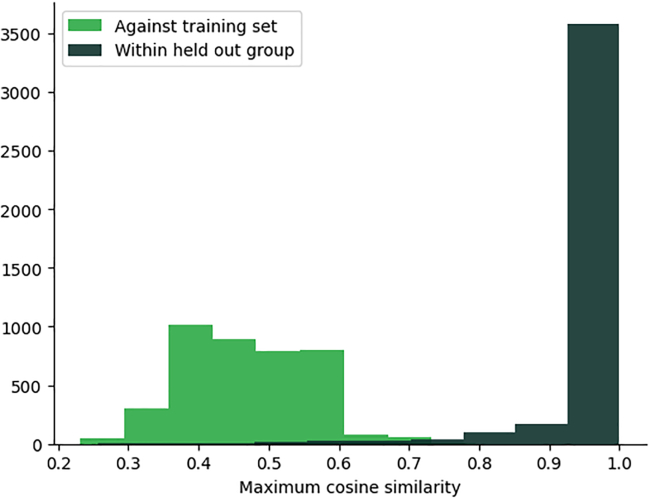
Cosine similarity of each of the HiFi-NN embeddings of each held out sequence to the training set and to each other

Multi-label classification is a challenging problem in any domain. In particular, the datasets used in this study for EC annotation have large class imbalances. We expect other machine learning based methods may excel at particular classes where our model may perform poorly and vice versa. The results we report on the benchmark datasets used in this study are aggregated across the entire dataset; future work could examine particular sequences for which methods differ in their predictions and why that may be the case. In additional resources we show that our method can be used to identify the third level EC for sequences which belong to a fourth level of EC classification not present in the training set. However, the models performance is quite weak in this instance. What is more interesting is that the embeddings of the sequences from the held out fourth level EC are all much closer to each other than the other sequences within the training set. This suggests potential for embedding models in the discovery process of novel fourth level EC numbers but is not something we explored further in the context of this study (Figures 11 and 12).

## Resource availability

### Lead contact

Further enquiries can be directed to Gavin Ayres, gavin@basecamp-research.com.

### Materials availability

No new materials were generated in this study. Further information and requests for resources should be directed to and will be fulfilled by the lead contact, Gavin Ayres.

### Data and code availability

• Data: One is a proprietary metagenomic database and thus is not publicly available. The datasets which have been curated from the public domain and used in this study is available at https://doi.org/10.5281/zenodo.12706810.
• Code: The code used in this study is available at https://doi.org/10.5281/zenodo.12706810 and at the Github repo https://github.com/Basecamp-Research/HiFi-NN.
• Further information and requests for code and data availability should be directed to the lead contact Gavin Ayres.

## Acknowledgments

The authors gratefully acknowledge the biodiversity stakeholders around the world who have granted Basecamp Research permissions to access their sites for the collection and analysis of environmental samples for metagenomic research and commercialization purposes.

## Author contributions

The method was conceived and developed by G.A. G.M., M.H., and K.Y. advised on dataset construction and evaluation. P.L., G.M., and N.F. proposed the case studies present in the paper. G.A., G.M., P.L., N.F., and B.B. contributed to writing the final manuscript.

## Declaration of interests

G.A., G.M., and P.L. are employees of Basecamp Research with B.B. being an employee of Basecamp Research at the time of writing. N.F. and K.Y. are scientific advisors to Basecamp Research. K.Y. is an employee of Microsoft Research. M.H. is an employee of Sanofi.

## Declaration of generative AI and AI-assisted technologies in the writing process

No generative AI or AI assisted technologies were used in the production of this work.

## STAR★Methods

### Key resources table

**Table:** 

REAGENT or RESOURCE	SOURCE	IDENTIFIER
**Software and algorithms**		

Datasets derived from Swiss-Prot	The Uniprot Consortium^28^	www.uniprot.org
BRENDA case studies	Rembeza et al.^24^	https://doi.org/10.1371/journal.pcbi.1009446
CLEAN	Yu et al.^17^	https://doi.org/10.1126/science.adf2465; https://github.com/tttianhao/CLEAN
ProteInfer	Sanderson et al.^21^	https://doi.org/10.7554/eLife.80942; https://github.com/google-research/proteinfer
DIAMOND BLASTp	Buchfink et al.^29^	https://doi.org/10.1038/s41592-021-01101-x; https://github.com/bbuchfink/diamond
NMPFams database	Baltoumas et al.^26^	http://www.nmpfamsdb.org
PyTorch	Paszke et al.^30^	https://pytorch.org
Scikit-Learn	Pedregosa et al.^31^	https://scikit-learn.org
NumPy	Harris et al.^32^	https://numpy.org
ESM-2	Lin et al.^4^	https://github.com/facebookresearch/esm
Code and datasets used for this study		https://doi.org/10.5281/zenodo.12706810

### Method details

#### Dataset construction

To construct training, testing and validation sets, all clustering was performed using the tool MMSeqs2^33^ using iterative profile search with the highest sensitivity setting (7.5) for identities below 50%, a coverage of 0.8 and default parameters otherwise. At each iteration we removed a set of clusters and added only the representatives of these clusters to the test set. We ensure the same EC labels exist in the training, validation and test datasets by removing sequences in the validation and test sets for which there is no sequence in the training set which has the same EC number. We assess the performance of HiFi-NN in diverse scenarios, and to do so, we assembled different datasets, each with a specific purpose.

First, we compare how our model performs at varying clustering identities to the current most widely used protein annotation tool, DIAMOND BLASTp, which requires several test sets representing each identity threshold. The exact composition of each test set is outlined in Table 1.The associated training set comprises a total of 163,632 sequences.

Second, we prepared a dataset to calibrate our choice of *k* nearest neighbors and distance thresholds at which our model should refuse to annotate, outlined in additional resources. We clustered Swiss-Prot to 50% identity and removed 3000 cluster representatives as the test set. As outlined in the previous paragraph, we then ensure that each EC number which exists in the test set has at least a single representative in the training set. This gives us a final test set size of 1,977 sequences covering 644 EC numbers and a training set size of 166,404 sequences covering 2,808 EC numbers (≈ 49% of the total number within Swiss-Prot).

Finally, we construct a larger lookup dataset. For this we use Swiss-Prot clustered to 30%, with 100 sequences removed as a validation set, later supplemented with sequences from a proprietary graph database to see how performance changes as we add sequences to the lookup set which the model has not been trained on. The full list of EC numbers covered by HiFi-NN is listed in the supplementary material. In total the label space spanned by HiFi-NN trained on this dataset consists of 5,703 EC numbers.

For each of the aforementioned datasets we train a separate model, one for each lookup set used. This ensures that at training time the model does not have access to sequences which have a sequence similarity to the training set which we aim to evaluate performance on.

The Price enzyme dataset^22^ consists of 149 sequences spanning 56 EC numbers.

One clear avenue for improving the performance of our method is to scale the dataset size. In particular as the effectiveness of the k-nearest neighbor method depends on the class boundaries defined by the lookup set, we sought to supplement our Swiss-Prot training set with a diverse subset of sequences derived from a proprietary metagenomic database. Sequences derived from this database were collected from 5 continents, spanning 60 percent of WWF biomes,^34^ a pH range from 1.5 to 11.5, and a 108° C temperature range. Crucially, all sampling efforts were conducted with biodiversity stakeholder consent and engagement as well as landowner permission, following the access and benefit sharing guidelines & regulation for digital sequence information (DSI) as outlined in the Nagoya Protocol.^23^ These sequences, of course, need labels. To this effect we use the HiFi-NN model trained on Swiss-Prot to assign EC numbers to these sequences. We only assign annotations if the cosine distance between the sequence to be annotated and it’s closest hit in our training set is less than a certain threshold (see additional resources). Furthermore, to strengthen our confidence in the assigned labels we also use DIAMOND BLASTp with Swiss-Prot as the lookup set to annotate the same set of sequences. We then keep the labels for which this ensemble of methods have agreed.

There is no overlap between any of the test sets used and the training sets for each model against which they are benchmarked.

#### Contrastive learning with the EC hierarchy

We wish to map ESM-2 650M^4^ embeddings to a feature space in which distances between protein sequence embeddings reflect the similarity between their EC numbers, as defined by the EC number hierarchy. In order to do so we employ a contrastive learning approach, forcing similar sequences closer together and dissimilar sequences further apart. In particular, we operate on the residue level embeddings from ESM-2 650M,^4^ which are of dimension (l,1280) where *l* is the length of the sequence.

To facilitate an informative measure of similarity between labels, which also leverages the inherent hierarchy of the Enzyme Classification numbering system, we represent each EC number as a set with 4 elements. For example, take the EC number *EC 2.3.2.27* which represents the enzyme RING-type E3 ubiquitin transferase. We represent this as the set {‘2’, ‘2.3’, ‘2.3.2’, ‘2.3.2.27’}. Likewise, the EC number *EC 2.3.2.31* is represented as the set {‘2’, ‘2.3’, ‘2.3.2’, ‘2.3.2.31’}. We can compute some similarity measure between these sets and require the cosine similarity between the embeddings of sequences which have these EC numbers to somehow reflect this set similarity. The one we choose here is the overlap coefficient. In this example the overlap coefficient between the two sets representing *EC 2.3.2.27* and *EC 2.3.2.31* is 0.75, indicating that the first three levels of their respective EC number’s agree. This example is illustrated in Figure 2.

We minimise the mean squared error between the pairwise cosine similarities of all the samples in a batch and a pairwise set similarity between their associated labels. Specifically, we compute the loss, *L*, over the upper diagonal elements of the two pairwise computations.

(Equation 1) $$L=\sum_{i=1}^{i<j}\sum_{j=2}^{\left|B\right|}{\left(x_{i,j}-y_{i,j}\right)}^{2}$$

The similarities we use are as follows:

(Equation 2) $$\begin{array}{c} x_{i,j}=\frac{f_{\theta }\left(x_{i}\right)\cdot f_{\theta }\left(x_{j}\right)}{∥f_{\theta }\left(x_{i}\right)∥∥f_{\theta }\left(x_{j}\right)∥}\\ y_{i,j}=\frac{\left|\text{labels}\left(i\right)\left|\cap \right|\text{labels}\left(j\right)\right|}{\min\left(\left|\text{labels}\left(i\right)\right|,\left|\text{labels}\left(j\right)\right|\right)} \end{array}$$

These represent the cosine similarity, $x_{i,j}$, between the embeddings of a pair of sequences within a single batch and the overlap coefficient, $y_{i,j}$, between their respective sets of labels. The neural network with which we embed all of the sequences in the batch is represented by $f_{\theta }$ and the batch size is denoted |B|.

#### Architecture and training details

The architecture we use is a single transformer layer, to process the residue level embeddings, followed by a two-layer multi-layer perceptron (MLP) with an input dimension of 1280, a hidden dimension of 1024 and output dimension of 512. The transformer layer uses 20 query, key and value heads with a feedforward embedding dimension of 5120 and an embedding dimension of 1280. We use layer normalisation following the transformer layer and in the penultimate layer of the MLP. The activation function used is the Gaussian Error Linear Unit (GELU).^35^

Each Swiss-Prot model is trained on 4 NVIDIA A100 GPU’s until convergence on a hold out validation set. The model trained on Swiss-Prot clustered to 30% identity is trained for 150 epochs. Both the models trained on Swiss-Prot clustered to 50% and 90%, with clusters iteratively removed in increments of 10% identity thresholds, are trained for 50 epochs. Each model is trained with a batch size of 16 using the AdamW optimiser^36^ with a learning rate of 0.001 and default parameters otherwise. The learning rate is decayed according to a cosine annealing schedule to a minimum of 0.00005.

The nearest neighbor classifier forms the prediction set by transferring the labels of examples within the training set which are closest in space to the query. The metric we use to define closeness is cosine similarity. For the ESM-2 based nearest neighbors classifier we mean pool the embeddings of each sequence in our training set and store them in a flat index structure to facilitate exact nearest neighbor search using the FAISS library.^37^ For the HiFi-NN based nearest neighbor classifier we follow exactly the same procedure except we project the ESM-2 embeddings to a lower dimensional subspace using a neural network trained such that the cosine similarities between points in the subspace correlate with the similarity between EC classes.

#### HiFi-NN computational requirements

The nearest neighbor search is implemented using the FAISS library.^37^ For the most accurate search results we use the brute force implementation of nearest neighbor search with a flat index. This has time complexity of O(nkd) where *n* is the number of sequences in the training set, *k* the number of neighbors being searched for and *d* is the dimensionality of the feature space.

The expected-time computational complexity of BLAST is approximately $aW+bN+\frac{cNW}{20^{w}}$, where W is the number of words generated, N is the number of residues in the database and a, b and c are constants. In practice this can be approximately linear in the number of residues in the search database due to heuristics used to reduce unnecessary alignments.^29^

There are many improvements which can be made to the nearest neighbors algorithm and the method used to index the embeddings which can lead to dramatic speedups in search time, surpassing that of DIAMOND BLASTp. Improvements include approximate search methods such as Locality Sensitive Hashing (LSH) or the Hierarchical Navigable Small World (HNSW) and the Inverted File Index (IVF) with Product Quantization (PQ). We chose FAISS^37^ because these optimisations are readily supported by the software package as well as offering support for GPU based nearest neighbor search. As part of future work we will evaluate the performance of the model when approximate nearest neighbor search algorithms are used to perform the search.

### Quantification and statistical analysis

#### Metrics

We evaluate the model using precision, recall and F1 score. The recall of a classifier on a particular dataset is defined as:

(Equation 3) $$recall=\frac{TP}{TP+FN}$$

The precision of a classifier on a particular dataset is defined in the following way:

(Equation 4) $$precision=\frac{TP}{TP+FP}$$

Finally, the F1 score of a classifier on a dataset is defined:

(Equation 5) $$F1=\frac{2*TP}{2*TP+FP+FN}$$

Unless stated otherwise all metrics are computed on the 4th level of the EC number classification system. For example, Uni-Prot entry Q73SE4 has the associated label ‘2.7.7.6’ (DNA-directed RNA polymerase) which is one-hot encoded when we compute the metric. A prediction of ‘2.7.7.7’ (DNA-directed DNA polymerase) is considered incorrect.

### Additional resources

#### Related work

##### EC number classification

The task of assigning multiple labels to a test instance can be tackled in many different ways. DeepFRI^38^ uses a neural network architecture with a softmax output layer and is trained in a supervised manner. The authors attempt to alleviate the issues that come with an imbalanced dataset by using a weighted binary cross entropy loss function. Similarly, ProteInfer^21^ is trained in a supervised manner and therefore faces a similar problem. The authors of CLEAN^17^ showed that ProteInfer failed to maintain predictive power for underrepresented classes. DeepECtransformer^39^ also treats the task of EC annotation as a supervised learning problem, however unlike ProteInfer they include an unsupervised pre-training step in their method. The class imbalance is addressed by the use of a focal loss function. ECPred^40^ opts for an alternative method using an ensemble of classifiers, one for each 4th level EC number. However, coverage only extends to 858 EC classes, a problem not faced by the current most widely used annotation tool, DIAMOND BLASTp.^11^ HECNet^41^ incorporates the inherent hierarchy in the EC labeling system by including a hierarchical triplet loss as an inital training loss upstream of a feedforward neural network with a softmax loss function. Our method is similar in the use of a triplet loss, however we benefit from the use of ESM-2 650M^4^ as the pre-trained sequence encoder. CLEAN,^17^ attempts functional annotation of protein sequences by pairing an optimised feature space with a nearest neighbors classifier. This is a step toward deep learning based functional annotation which can handle class imbalance and the authors showed that for certain datasets it significantly outperforms DIAMOND BLASTp. Building on the work of^17^ we also use a nearest neighbors classifier on an optimised feature space to annotate protein sequences. Our method differs from^17^ in how we represent the classes and the contrastive loss we use to produce our feature space. Rather than a set of class prototypes (embeddings representing a single EC number) we use each example in the training set as an example of their associated class. This has a few practical benefits; it is straightforward to incorporate new EC numbers into our representation, a practitioner can choose to trade off precision and recall at inference time by varying the choice of *k*, and we can incorporate label density information into our confidence estimates. Furthermore, we can extend the size of our lookup set without the need for re-training. Searching over the entirety of the training set can be handled by the use of approximate nearest neighbor algorithms, e.g., FAISS.^37^ Recent works have sought to also approach the task of EC classification as a retrieval problem, for example^42^ use a retrieval-augmented classifier for assigning EC numbers to seqeunces. This method is not used as a comparison in this work due to absence of publicly available code.

The method we propose is similar in spirit to DIAMOND BLASTp and has been shown to be effective for annotating GO terms^43^ and CATH annotations.^20^ We transform a ’lookup’ dataset to our optimised feature space and then perform a nearest neighbor search against this lookup dataset. The annotations of the k nearest neighbors are then transferred to the query protein. Importantly, we only need to transform the training set once. Then at inference time we embed the query sequences and perform a *k*-NN lookup against the already embedded training set.

##### Deep metric learning

Contrastive learning involves comparing examples to each other and imposing a loss such that similar examples will be close in feature space and dissimilar examples far away. It has proven an effective tool for representation learning and has led to state-of-the-art performance on several image classification and text classification benchmarks as well as providing a means of aligning the feature spaces of data from multiple modalities.^44^

Contrastive learning has proven particularly useful in aligning the embedding spaces of different modalities,^44^^,^^45^ learning representations for labeled examples^46^^,^^47^ and for unsupervised representation learning.^48^ The typical approach is to design a loss function where examples from the same category are pushed close together and examples from different categories far apart. The approach we take is slightly different, we seek to make the or cosine similarity between two embeddings correlated with a similarity measure between their labels. To do so we simply minimise the mean squared error between the two.

##### Class probability estimates for multi label k-nearest neighbors

The relative distances between queries and neighbors has been optimised as part of the training process and so distance based confidence measures are a natural candidate. Conformal prediction provides a framework for computing such confidence measures through the use of non conformity scores calibrated to a validation set. These non conformity scores have been extended to the case of multi-label classification by considering the non conformity of the entire set of labels predicted for a given test instance relative to the power set of the labels.^49^^,^^50^ However, in the context of EC number annotation the powerset of all labels is simply too large to have practical application. The authors of^51^ show conformal prediction can lead to improvements in performance, but this is dependent on the calibration set used.^17^ makes use of statistical properties of the distances between and within EC numbers in order to calculate its confidence measure. This involves fitting a Gaussian Mixture Model to the distribution of within class distances and between class distances.

#### Performance relative to DIAMOND BLASTp

We compare our method to the annotation tool widely used for protein function annotation. We run the DIAMOND BLASTp^29^ version of the tool with default parameters and an e-value cutoff of 1e-3 with the highest sensitivity setting, *–ultra-sensitive*, so we achieve the best possible performance at low percent identities.

##### Extending into the midnight zone

To serve as a comparison to DIAMOND BLASTp and to illustrate the utility of protein language model embeddings in moving past homology based annotation at low sequence identities we created a dataset following the procedure used for the ProteinNet^52^ dataset. We cluster Swiss-Prot to sequence identities ranging from 10% to 90% in increments of 10, removing a set of clusters at each iteration and adding only the representatives of these clusters to the validation set. We choose clusters that have a minimum of 5 sequences to avoid validation sets which are composed entirely of protein fragments (typically very short proteins which have no homologs in the rest of the dataset) or mis-annotated sequences. The total number of clusters we remove from the training set at each clustering is 300, we then take one sequence from each cluster and add it to the validation set representing the chosen sequence identity. This gives us 9 validation sets with 300 sequences each, each sequence corresponding to an entire cluster. The remaining set of sequences comprises our training set. After clustering we found that there were 830 EC numbers across all our validation sets which did not exist in the training set. It would be impossible for any method to correctly annotate these EC terms and so we remove sequences corresponding to these EC numbers from our validation sets. This ensures we preserve the sequence similarity thresholds we desire. We have a total of 163,632 sequences in our training set. The resulting composition of our validation sets is outlined in Table 1 and the performance of each of these sets outlined in Figure 4. We use the training set as our lookup set for annotation. We report the sample average of each metric due to the differing test set sizes.

##### 50% sequence identity

The second training set we construct was designed to study the effect of a larger training set and a larger test set. We clustered Swiss-Prot to 50% identity and removed 3000 cluster representatives as the test set. As outlined in the previous paragraph, we then ensure that each EC number which exists in the test set has at least a single representative in the training set. This gives us a final test set size of 1,977 sequences covering 644 EC numbers and a training set size of 166,404 sequences covering 2,808 EC numbers (≈49% of the total number within Swiss-Prot). The performance of HiFi-NN with these dataset splits is outlined in Figure 9D). As before, we report the sample average of each metric. We do not compare against other deep learning based methods as we cannot ensure a bound on the sequence identity between the training set for each method and this test set.

#### Choice of *k* nearest neighbors

The effect of varying the *k* nearest neighbors on performance on the 50% identity cluster representatives dataset is outlined in Figure 3. It is worth noting that this is a choice made at *inference*. A practitioner can trade off precision and recall by varying the number of nearest neighbors they wish to retrieve. As a consequence, we provide a predicted probability score threshold for improving precision and recall for a fixed choice of *k*. The predicted probability score threshold takes into account both distances to labels and the density of a label amongst the *k* nearest neighbors. As such, it becomes more useful as *k* is increased, for k=1 it will trivially assign a predicted probability of 1.0 to all annotations. For lower values of *k* a distance based cutoff is recommended.

#### When not to annotate

To establish a threshold at which we refuse to annotate a protein sequence we use sequences from Swiss-Prot which have not been annotated with an EC number and have an annotation score of 5 (a measure of the reliability of the annotations associated with a protein, on a scale of 1–5). The results are illustrated in Figure 8B). The lookup set used for this study is the validation set derived from the 30% clustering. We take the 75th percentile of the distances to the test set which has an EC, a distance of 0.38.

#### Class probability estimates for multi-label k-nearest neighbors

The practical utility of an annotation tool necessitates a reliable confidence score or estimated probability of the assigned labelset being correct. To this effect there are two broad categories of approaches which we may pursue, scores based on distances from a query to an example, with associated labels, and scores based on the density of labels in the neighbors.^53^

The approach we opt for uses information from both the density of the labels amongst k nearest neighbors as well as their associated distances. Specifically, we extend probability density estimates for local neighbourhoods^54^^,^^55^ to the multi-label setting. The setup is as follows; we have a set of real valued vectors, $x_{1},\ldots ,x_{n}\in R^{D}$ and a finite set of labels Y. The aim is to learn a classifier which maps from the input space X to the powerset of the labels, assigning scores in proportion to the relevance of a given label to the test data point. For an instance, *x*, and its associated labelset $Y\subseteq 2^{Y}$ we will denote the neighbors of *x* using N(x) and a distance vector for each label *l* as

$${\vec{y}}_{x,t}\left(l\right)=\left\{ \begin{array}{cc} d\left(t,x\right), & \text{if}\;l\in Y\\ 0, & \text{otherwise} \end{array}\right.$$

where d(t,x) is a distance metric between two vectors $t,x\in R^{D}$. We then define the probability of an instance *t* having label *l* as a softmax over the distances between *t* and each instance in the neighborhood of *t* with label *l*,

(Equation 6) $$p_{t,l}=\frac{\sum_{x\in N\left(t\right)}e^{-{\vec{y}}_{x,t}\left(l\right)/T}}{\sum_{x\in N\left(t\right)}e^{-d\left(t,x\right)/T}}\text{,}$$

where *T* is a scaling parameter which controls the relative influence of nearby points. We set T=0.001 for all experiments.

In Figures 6C and 6D, we see further validation that increasing the sequence diversity of the training set improves the method. The median confidence score for all correct predictions changes from 0.14 to 0.28. The model becomes more confident in its correct predictions, this is likely due to the fact that our confidence measure is based on the cosine distance to each neighbor and the relative density of a label within the *k* nearest neighbors. Increasing the number of sequences per EC class and the diversity of such sequences will help on both fronts.

#### Performance vs. diversity

As outlined in the results 2.4 the addition of extra sequences seems to have no effect on the performance of the model on the time-based split of Swiss-Prot. One simple explanation for this may be simply due to the sequences we have added not being close in sequence space to these test sequences. This difference is elucidated in Figure 7.

In fact, if we look at the distance between each sequence in the Price-149 dataset and its closest hit in each of our training sets we see the reverse pattern, Figure 7B). Future studies could examine the diversity in sequences needed per class to learn a representative embedding space. Similarly to CLEAN each class could be represented by a single prototype, in this paper we have represented each class by a number of prototypes (the number of sequences we have per class in our training set). However, similar performance should be possible with a reduced number of prototypes per class, perhaps based on the embeddings of a non redundant set of sequences for that particular EC class.

#### Performance on hold-out classes

To test how well the contrastive learning approach captures the label hierarchy we trained a model with certain 4th level classes excluded. We exclude EC numbers 2.7.4.4 and 2.7.7.6. These are the two classes for which we have the fewest, 1, and most, 3969, examples respectively. The model is trained for 50 epochs with a batch size of 16 using the AdamW optimiser^36^ with a learning rate of 0.001 which is decayed according to a cosine annealing decay schedule to a minimum of 0.00005.

In Figure 11 we show the performance of the model at annotating the third level class correctly on this hold out set. To evaluate performance we truncate each class annotation to the third level of EC number, so 2.7.4.4 becomes 2.7.4 and 2.7.7.6 becomes 2.7.7., and measure performance as discussed in section metrics.

We also wished to see if the model embeds each of the hold out sequences in a similar region of space to each other. To test this we compared the maximum cosine similarity of each member of sequences in our hold out set 1) to each other and 2) to the training set. Not only do we see, in Figure 12, that the within group cosine similarity is higher than the hits each sequence has in our lookup set, we also see that a significant proportion share a cosine similarity greater than 0.9 indicating they may share a fourth level EC number. It is important to remember that the model has not been trained on the fourth level EC number that these sequences represent.

## Supplemental information

Table S1. This table lists all of the EC numbers covered by the HiFi-NN annotation tool
